# Perspective of trust towards e-government initiatives in Sri Lanka

**DOI:** 10.1186/s40064-015-1650-y

**Published:** 2016-01-07

**Authors:** H. M. B. P. Ranaweera

**Affiliations:** School of Public Administration, Huazhong University of Science and Technology, Wuhan, People’s Republic of China; Department of Business Management, Rajarata University of Sri Lanka, Anuradhapura, Sri Lanka

**Keywords:** E-government, TAM, Trustworthiness, Structural equation modeling

## Abstract

In this article, the author focuses on understanding the antecedent conditions of trustworthiness of the users towards the use of e-government services and attempt to propose a model to assess the influences of the trustworthiness for the use of e-government services in Sri Lanka. Trustworthiness was considered as an additional construct along with the technology acceptance model (TAM) constructs. Structural equation modeling (SEM) approach was used to test the proposed model by utilizing the responses of 898 citizens. SEM results reveal that the proposed model is acceptable showing goodness of fit. The proposed model tested by SEM is appropriate to assess what extend trustworthiness of the users influence for the use of e-government services and this would be worth to pay more attention on trust when develop and implement e-government initiatives.

## Introduction 

Delivering government services have been changed with the adoption of Information and Communication Technologies (ICT) (Colesca and Dobrica 2008). Governments use the power of ICT to boost the government services and improve services spending huge money (Ahmed 2013; Lee 2010; Alshehri and Drew 2010). Meanwhile, the power and the capabilities of the people also have increased with ICT (Christos et al. 2013; Syamsuddin 2011). Accordingly, people expect same experience which has gained from private sector from government sector too (Evans and Yen 2005; Henkel et al. 2014). Thus, utilization of ICT is indispensable to governments.

Despite the potential benefits of huge investment to e-government initiatives, most countries are facing to challenges related to acceptance, adoption and use (Akkaya et al. 2012; Shajari and Ismail 2013). Although, these issues are much more explored, some other aspects are yet to be investigated (Rana et al. 2015b). Extant literature reveals that user trustworthiness shapes the feelings of the people and hinders the adoption and use of e-government services (Rana et al. 2015b). Simply, trustworthiness implies as keeping confidence that one’s promises can be relied (Rotter 1971). The significance of trustworthiness of users has been pointed out by many scholars as well as this particular factor make significant influence on adoption and use of e-government services (Alshihi 2005; Carter and Weerakkody 2008; Lean et al. 2009; Srivastava and Teo 2009; Akkaya et al. 2012; Sharma et al. 2014; Zhou 2015) and this will be a barrier to accept and adoption of e-government services. However, this particular significant factor has not been explored much more in the context of e-government (Srivastava and Teo 2009; Karunasena and Deng 2009; Alaaraj and Ibrahim 2014) further, it is noted that the importance of trust in an online business environment has been explored tremendously (Lee et al. 2015), but it is few in the context of e-government. Accordingly, trustworthiness plays a crucial role for acceptance, adoption and use of e-government services in a country (Rufin et al. 2014; Taiwo et al. 2014). According to the investigation done by Rana et al. (2015b) titled A meta-analysis of existing research on citizen adoption of e-government, it was revealed that no proper study has done in the context of Sri Lanka. Hence, this study recognizes trustworthiness as an influencing factor on use of e-government services and tries to understand the antecedent conditions of trustworthiness and suggest a model to answer the question of Does trustworthiness influence the use of e-government services in Sri Lanka?. The objectives of this study are to identify the antecedent conditions of trustworthiness and propose a conceptual framework that can serve as a base model which is worth to assess the influence of the trustworthiness for the use of e-government services in Sri Lanka.

The paper is organized as follows. This section includes the foregoing introduction. The next section provides background of the study following sub sections of antecedent conditions of trustworthiness and theoretical background of the study. Next, presents the proposed model in section three. Section four is devoted to discuss the methodology applied to the study. The model fit for the proposed model is presented in section five and final section brings the paper conclusion.

## Background of the study

Country of study is Sri Lanka being a representative South Asian country having the 1st place in the e-government development index of the region and 74th in the world (United Nations 2014). With regard to the culture, different nations have their own unique culture and form of the trustworthiness of the people also differs across the nations (Hofstede et al. 1990). So that, people’s behavior on trustworthiness connected to e-government services changes according to nations. Accordingly, the studies which have been addressed to the trust issues in developed countries are not well suited to explore the issues related to trustworthiness of the users in a country like Sri Lanka. People are reluctant to use and obtain services from e-government websites due to the lack of trustworthiness towards the government websites (Belanger and Carter 2008). Unlike traditional brick and mortar government services, e-government services are unique and impersonal in nature; hence, issues related to trustworthiness are raised with technological advancements. The infrastructure and platform used in these e-government websites are related to open technologies such as internet. So that, people wants an assurance when they involve in so called e-government websites. Accordingly, e-government services will only be embraced by people if people feel trustworthiness is secured in government websites (Belanger and Carter 2008).

In the absence of trust, people demotivate and avoid from e-government websites and move to traditional brick and mortar government services (Teo et al. 2008) further, highlighted as trust is a crucial determinant to retain users towards the e-government websites. Trust has been discussed and has defined in different ways. Trust is defined as *expectancy that the promise of an individual or group can be relied upon* (Rotter 1971). Another way, trust has defined as the understanding of the confidence in the online sellers’ reliability and integrity for exchanging goods or services electronically (Belanger et al. 2002). According to both definitions, it implies that trustor keep hopes about the trustee who will not break the given promise. Simply, it can be recognized as keeping confidence that particular desired party do not do bad or harmful things. In this scenario, trustworthiness has identified from the e-government users’ point of view. Trustworthiness makes people relaxed for giving personal information and obtaining services in a virtual environment such as e-government and this trend leads to use e-government services continuously. Commercial websites could have been preserved the trustworthiness of the users especially in online transactions and this leads to success of their e-commerce. Accordingly, success of the e-government websites is also determined by the user’s level of trust (Teo et al. 2008). E-government websites are the substitute channel to offer government services instead of using traditional offline channels. So that, the government should assure that the government websites are functioning effectively and able to offer services quickly with minimum cost involving. If so, people will be motivated to embrace e-government services with positive believes that government websites have developed and maintain to serve their needs and ultimately this will form trust among the user and tends to use continuously.

This study is based on the emerging area of interest related to trustworthiness of the users towards the use of e-government services in Sri Lanka. In line with this thinking, researchers have built up arguments on trustworthiness and have shown a significant positive relation between trustworthiness and use of e-government services (Teo et al. 2008; Colesca and Dobrica 2008). Thus, prior evidences focusing to identify the antecedent conditions which form the trustworthiness on the use of e-government services are presented in next sub section.

### Antecedent conditions of trustworthiness

Trustworthiness is an expectation on other party who does not behave opportunistically to take the advantages of the particular situation (Lee et al. 2015 cited from Gefen et al. 2003). Furthermore, they have proposed trust as “cognitively active construct”. Trustworthiness is a key issue especially in an online environment where as more uncertainty can be seen (Hajli 2015 cited from Pavlou 2003). To endorse trustworthiness in an e-government context, it is necessary to pay more attention to build up trust among the users having some strategic mechanisms by emphasizing the credibility of the government websites. On the other hand, trustworthiness encompass people’s willingness to get information and give information in which government website ask from the people and do the transaction accordingly. Prior studies give evidence that lack of trustworthiness is a major barrier to acceptance and adoption of e-government services (Carter and Weerakkody 2008). Accordingly, it should be identified that what kinds of antecedents are needed to build up trustworthiness. Through the literature, it was revealed that trustworthiness is formed with different dimensions and it can be considered as multi-dimensional construct (Bianchi and Andrews 2012).

Scholars have explored this particular construct in the online environment as well as in the context of e-government. They have identified and shown the importance of different dimensions which are applied in various situations and environments. Thus, reviewing past studies, it was able to identify five significant dimensions which cause to build the trustworthiness in the context of concerned country. The acknowledged dimensions are; Trust in government and internet (Belanger and Carter 2008; Teo et al. 2008; Mofleh and Wanous 2008; Mahadeo 2009; Akkaya et al. 2012; Sharma et al. 2014; Alomari 2014); e-government is an internet-driven activity like other innovative services such as e-commerce, e-business, e-banking. Thus, users of self-service technologies like e-government concern about reliability of the websites. They expect to complete the desired task successfully in the virtual environment without facing technical problems. If they face such issues their trust on government websites may drop. This implies that service provider should set up the structural conditions (related to technology) to complete a task without an error. Those who have trust in the internet are easily adapted to e-government services as well as those who trust the government also adapt easily. Mahadeo (2009) has considered trust of the government and trust of the internet as one dimension, since these two go with together. Accordingly, trust in government and internet is treated as one dimension for this study too.

Perceived security (Connolly and Banister 2006; Kim et al. 2008; Beldad et al. 2012; Ayyash et al. 2013; Ahmed 2013; Al-Jaghoub et al. 2010; Sadeghi and Farokhian 2010; Liu and Zhou 2010); also plays a big role for building the trustworthiness of the users toward e-services and less security act as a barrier for e-government adoption and use. Thus, perceived security enhances the trust level of users in e-government. Security issues should be taken into consideration when build up trustworthiness among the people with regard to e-government services. Perceived privacy (Ahmed 2013; Reay et al. 2013; Sadeghi and Farokhian 2010; Beldad et al. 2012); privacy issues may involve loss of personal information, misuse personal details etc., if users feel privacy problems when they do transactions or providing their personal information to government websites, immediately they avoid using. Hence, privacy issues should be addressed to form trustworthiness in self-service technologies. Perceived uncertainty/risk (Belanger et al. 2002; Schaupp and Carter 2010; Shareef et al. 2011; Akkaya et al. 2012; Bianchi and Andrews 2012; Rana et al. 2015a); risk refers to trustor’s feelings about the possibility of receiving gains or losses and perceived risk is a significant antecedent of trust. Involved risk should be less to increase the level of trustworthiness of users for e-services. Normally, the risk involved in online environment is high and this will be a barrier to adoption of e-services. Schaupp and Carter (2010) noted that risk is involved with uncertainty and twisted with each other. Thus, uncertainty and risk was considered as one dimension of trustworthiness for the present study.

Information quality (Lee et al. 2015; Ayyash et al. 2013; Akram and Malik 2012; Teo et al. 2008; David et al. 2004; Almahamid et al. 2010); content of a website should match with the user requirements. What user expects from a particular website should be able to fulfill. So that, if the content is relevant to user requirements user will be satisfied and retain and content relevance implies information quality. The user’s judgment about the available information depends on information quality. If the information in government websites is accurate, valid and timely updated, appropriate for their tasks, comprehensive, relevant, error free and precise, it is said that quality is there. Accordingly, if people feel that quality information can be obtained through government websites, then people’s confidence will be built up and it leads to make trustworthiness about the use of e-government services. Above evidences reveal that information quality makes trustworthiness of user and considered as a dimension for the study.

As mentioned in the above, it is revealed that scholars have been emphasized that these dimensions have relationships to trust and trust leads to take decision for adopting and use of e-government services. However, they have not used these dimensions together in one empirical study. Prior evidences indicate that different studies have used different dimensions. Thus, it is important to consider all identified dimensions together to assess the trustworthiness of users. Consequently, for the present study, researcher considered these five dimensions of trustworthiness and suggested following mentioned model with TAM constructs.

### Theoretical background

E-government initiatives are the products of information technology (IT) and considered as technological innovation (Teo et al. 2008). Consequently, behavioral adoption theories are suitable enough to know about the adoption of e-government. Thus, empirical studies related to the topic of e-government have done much more with the various technology adoption theories in order to improve user acceptance and adoption. Used theories are theory of reasoned action (TRA) (Fishbein and Ajzen 1975), theory of planed behavior (TPB) (Ajzen 1991), diffusion of innovation theory (DOI) (Rogers 1995), technology acceptance model (TAM) (Davis 1989) and unified theory of acceptance and use of technology (UTAUT) (Venkatesh et al. 2003). Extant literature gives evidence that TAM is the most cited and applicable in technology adoption research (Rana et al. 2011). Moreover, it has been rewarded through validation (Zha et al. 2013; Shajari and Ismail 2013; Aggorowati et al. 2012). On the other hand, some important factors such as trust and culture have not been considered with the TAM (Abbasi et al. 2015; Shareef et al. 2011; AlAwadhi and Morris 2008). Voutinioti (2013) has pointed out that though the prior studies give various models which are noted above may not be valid for all the cases in the technology adoption field. Researchers face confusions on selecting and deciding suitable and appropriate model among multitude of models. Because, acceptance and adoption of e-government is determined by various factors which differ among various user groups in different culture, level of use and interaction and commitment paid by the particular government and people.

## Proposed model

**Fig. 1:**
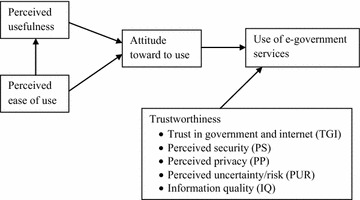
Proposed model

E-government initiatives are one of technological innovations found due to ICT (Teo et al. 2008). On the one hand, e-government websites are much more than ICT and e-government websites should attract the peoples’ attention and for the continuous use. As intimated above, literature (Belanger and Carter 2008; Ayyash et al. 2013; Beldad et al. 2012; Liu and Zhou 2010; Alomari 2014; Teo et al. 2008; Reay et al. 2013; Lee et al. 2015; Akram and Malik 2012; Mahadeo 2009) gives evidences that trust in government and internet, perceived security, perceived privacy, perceived uncertainty/risk and information quality are prevailed with websites and cause to form trustworthiness toward e-government. So that, trustworthiness is a vital construct which has the power to attract and retain users towards the e-government websites which no other alternative websites offer same services to people. The importance of adding trust construct to e-government adoption model has been shown by Voutinioti (2013). He has modified UTAUT model by adding trust construct in his study and stated that people’s trust would influence positively the use of e-government services and uncertainty also plays a big role in the e-service environment. Hence, uncertainty situations should be removed from the e-government website environment. As mentioned above, according to the prior literature, several adoption models can be identified, one of the models is well known and frequently use in information system adoption research as well as in the studies related to e-government. Technology acceptance model (TAM) is the one found by Davis (1989). Later, this model was modified and present as TAM2 (Venkatesh and Davis 2000), TAM3 (Venkatesh 2000). Lai and Li (2005) noted that TAM model has been used by researchers to empirical investigations and has achieved great success with survey approach. Moreover, they posit TAM is a mature model with validated in different context. But they have highlighted TAM still need to be tested for different context such as various respondent sub groups in different cultural environment. AlAwadhi and Morris (2008) and Shareef et al. (2011) revealed that those models are not enriched with some critical variables like culture and trust. The theoretical background of the present study is based on the TAM which has not addressed trustworthiness. Accordingly, TAM was extended considering identified dimensions of the trustworthiness and Fig. 1 illustrates the proposed model of the study.

Based on TAM constructs and dimensions of the trustworthiness, the theoretical model is proposed for assessing the effect of trustworthiness for the e-government initiatives in Sri Lanka. Dependent variable of the model is the use of e-government services (Useofe-Gov). According to TAM, attitude toward to use (A_use) is determined by perceived usefulness (PU) and perceived ease of use (PEU). Use of the e-government service is determined by attitude toward to use and trustworthiness. The proposed model should be validated to find out the suitability in the context of e-government initiatives in Sri Lanka.

## Research methodology

Deductive approach and survey strategy were used for the study. Survey strategy allows the researcher to collect quantitative data at a large scale (Saunders et al. 2009). This study used quantitative approach and numerical data was collected by administering a structured questionnaire. It is easy to inference by applying statistical techniques with quantitative data than the qualitative (Shajari and Ismail 2013). Structural equation modeling was applied for the analysis.

### Using structured equation modeling

The use of structural equation modeling (SEM) in research has been increased in many study areas such as psychology, sociology, education, political science, education and economics etc. (Khine 2013; Lei and Wu 2007; Anderson and Gerbing 1988). Testing the structural relationships in compliance with existing research theories and findings is the goal of the SEM and is one of the modern statistical approaches to fit the models with confirmatory factor analysis (CFA). Multiple variables [exogenous—represents simply independent variables, endogenous—represents simply dependent variables (Garson 2008)] can be handled simultaneously and effectively with more confidence in SEM (Byrne 2010). The proposed model based on literature review need to be tested against the collected measures items using CFA. The model fit indices in CFA will assist to determine whether the model is well fitted with data or not. The fit indices’ values are compared with cut-off values suggested by (Hair et al. 2006; Anderson and Gerbing 1988). This process ensures the reliability and validity of the model and the model fit, afterwards can go for assessing relationships between variables.

### Quantitative study

Structured self administered questionnaire was used to collect the data to test the proposed model. The survey items were selected from previous studies and some were modified accordingly to the research aim and the country context. The questionnaire consists with four sections. First section covers the basic demographic characteristics such as age, gender etc. the second section focus to get some idea about the experience regarding internet and government websites. The survey items related to variables were covered in both sections three and four. The five point likert scale ranging from 1 “strongly disagree” to 5 “strongly agree” are used to record the level of agreement for non-demographic survey items.

A pilot study was conducted in order to clarify the understanding or any discrepancies of the survey items. 30 respondents were asked to fill the questionnaire and convenient sampling was applied. Through the pilot survey, it was able to identify that this questionnaire is easy to answer. Only complain raised was about the length of the questionnaire, modification was made accordingly. It was revealed that all inter correlations exceed 0.30 for all items and item to total correlation exceeds 0.5 showing acceptance for continuing the data collecting. Cronbach’s alpha values were calculated and values exceed the threshold value 0.70 (Hair et al. 2006).

The data collection was occurred from six categories of the respondents reside in five provinces selected by following multi stage procedure. Undergraduates (university final year students), academics (university teachers), government employees, private sector employees, businessmen and normal civilians were included into six categories. An adequate sample size is necessary to be confident about the result and for better generalization. In line with that idea, 600 university students, 200 academics, 300 government employees, 300 private sector employees, 100 businessmen, and 100 normal civilians were selected conveniently as a sample for the study. Accordingly 1600 questionnaires were distributed and 1008 were returned, which out of these, 898 were valid for the study recording 56 % responding rate. After finishing collecting data, all entered into SPSS 16.0 data sheet for the preliminary analysis. AMOS 20.0 was used for further analysis.

### Reliability, validity and measurement items analyses

Internal consistency of the measurement items is very important to maintain the quality of the output of the research (Sekaran 2003). Homogeneity of the measurement items is indicated by the internal consistency of measures. Accordingly, Cronbach’s alpha values were calculated for each constructs and alpha values shown in Table 1 are acceptable with the cut-off value 0.7 (Hair et al. 2006). In addition to the calculation of alpha values, factor analysis was applied to assess the construct validity. Kaiser–Meyer–Olkin (KMO), average variance extracted (AVE) and construct reliability (CR) values are also considered to determine the construct validity. KMO value rages from 0 to 1, which higher the KMO value, it is said that more correlating factors represent the particular variable. Thump-rule of the KMO is >0.5. The Table 1 shows the calculated KMO values for the constructs of the study and all values exceed the cut-off 0.5. The cut-off values for AVE and CR are 0.5 and 0.7 respectively. Higher value suggests better validity. AVE and CR values are shown in Table 2. Values suggest that sufficient construct validity is there to proceed further analysis.

**Table 1:** Cronbach’s alpha coefficients for constructs

Constructs	No. of items	Cronbach’s α	KMO values
Use of e-government services (Useofe-Gov)			
Search information (SI)	03	0.807	0.705
Contact via government websites (CON)	03	0.760	0.688
Willingness to use government websites (WU)	04	0.740	0.761
Attitude toward to use (A_use)	08	0.875	0.903
Perceived usefulness (PU)	09	0.863	0.895
Perceived ease of use (PEU)	09	0.840	0.857
Trustworthiness			
Trust in government and internet (TGI)	05	0.783	0.794
Perceived security (PS)	03	0.700	0.638
Perceived privacy (PP)	04	0.746	0.733
Perceived uncertainty/risk (PUR)	03	0.703	0.675
Information quality (IQ)	04	0.769	0.768

**Fig. 2:**
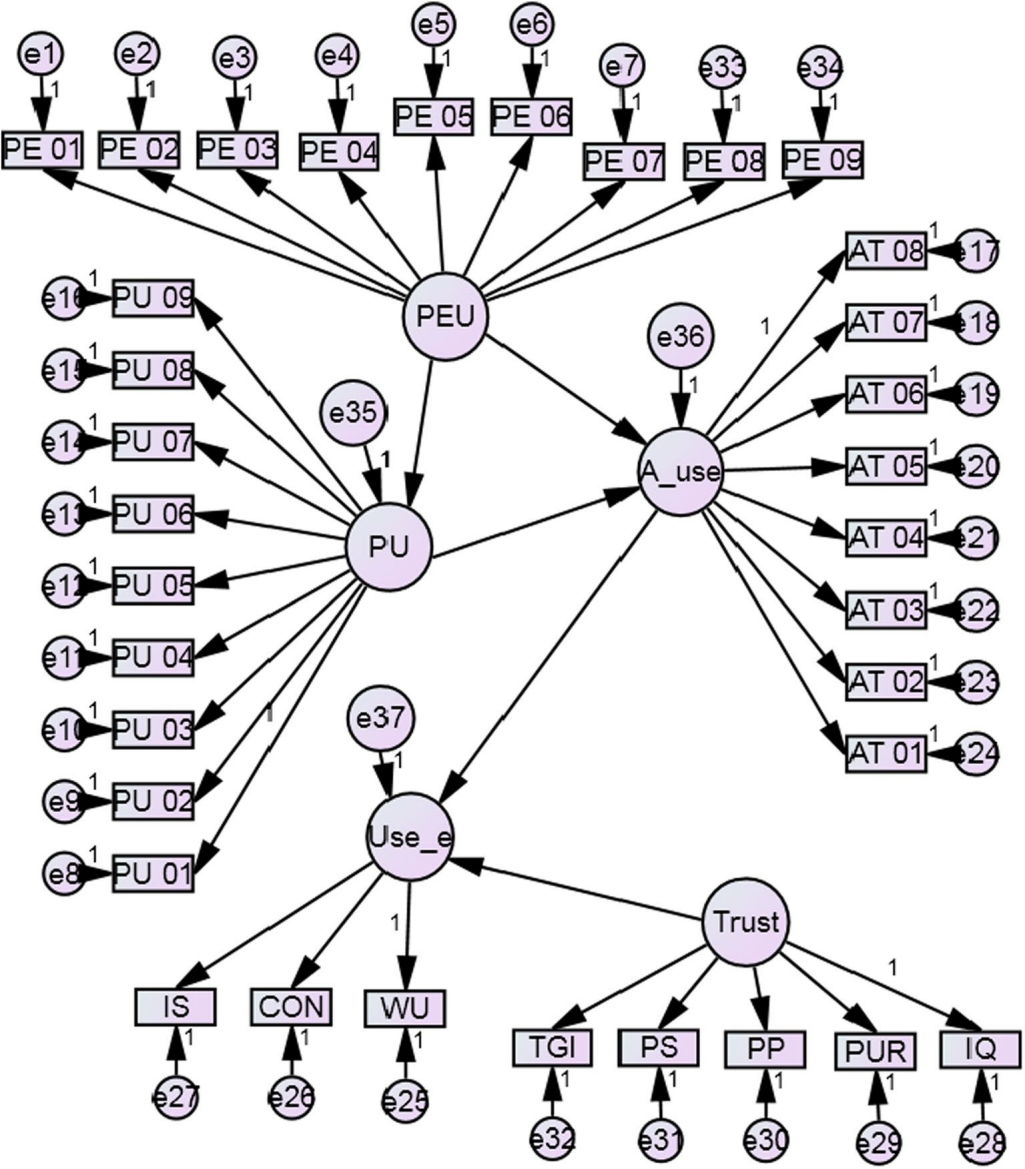
Initial proposed model

After finalizing measurement items for each variable, confirmatory factor analysis was carried out separately for each variable. This was carried out to identify any irrelevant items and to set the model fit for each construct separately. This procedure was supported to design the measurement model. Figure 2 illustrates the measurement model which was used for the factor analysis.

According to the CFA for each factor, it was revealed that normal Chi squares for all the factors except Useof-Gov were above five, which is not acceptable. For the good fit, values for the normal Chi square should be less than five (Hair et al. 2006). Byrne (2010) posits that it is hard to see a perfect fit between observed data and hypothesized model. Xiong et al. (2015) notes the same idea by mentioning that more possibility is there to have poor goodness of fit. Thus, modifications are necessary to have a better fit. Goodness of fit index (GFI) indicates the relative amount of variance and covariance of the model. The values of the GFI vary from 0 to 1 and higher values show the good fit. Adjusted goodness of fit index (AGFI) corrects the GFI value which is influenced by numbers of measurement items of each latent variable. Same as the GFI, higher value shows the good fit. Model fit is analyzed by comparative fit index (CFI). To do the analysis, it examines the inconsistency between data and the hypothesized model. Values of the CFI ranges from 0 to 1 and higher value indicates better fit. The discrepancy between the Chi squared value of the model and null model is analyzed by normed fit index (NFI) and for this index also, values range from 0 to 1 and value closer to 1 indicates good fit. Trucker Lewis index (TLI) resolves some sort of negative bias issues of the NFI. Like other indices, values rage from 0 to 1, with value closer to 1 shows good fit. Accordingly, values of fit indices; GFI, AGFI, CFI, NFI and TLI were matched with cut-off values. The value of these indices range from 0 to 1 and value above 0.9 indicates good fit (Hair et al. 2006; Hu and Bentler 1999). To solve the Chi square value mismatching, few modifications were made to each factor according to the suggestions from the modification indices.

For the factor of trustworthiness, the modification indices indicated a covariance between error term of PP and error term of IQ. Connecting these two error terms gave a better model fit without changing anymore. The modified model showed 2.098 normal Chi square (P value: 0.078) indicating good model fit. The fit Indies of GFI (0.996), AGFI (0.986), CFI (0.996), NFI (0.993), and TLI (0.991) were more than 0.9. Furthermore, root mean square error of approximation (RMSEA) was 0.035 and root mean square residual (RMR) was 0.007, which both RMSEA and RMR values should closer to zero for better fit a model (Hair et al. 2006). Consequently, modified trustworthiness model can be used for the proposed final model.

Perceived usefulness (PU) was the next model which needs to be modified for a better fit. According to the suggestions from modification indices of the CFA results, the covariance were recognized, which from e1 to e2, e4 to e5 and e8 to e9 and realized that can be correlated with each other. After the modification, model fit for PU is acceptable and suitable for final model. The normal Chi square is 4.756 (P value: 0.000), which is less than five. GFI (0.970), AGFI (0.944), CFI (0.996), NFI (0.959) and TLI (0.951) exceed 0.9. RMSEA (0.065) and RMR (0.025) also are below the cut-off values. Hence, model for perceived usefulness is acceptable.

Perceived ease of use (PEU) model was checked with CFA results and identified that the model is not fitted well. Thus, the modification indices were reviewed to have a better fit model. The modification indices show significant covariance, which can be merged (e1 to e2, e5 to e6 and e1 to e6). The modified model shows acceptable model fit. The values for GFI (0.952), AGFI (0.910), CFI (0.935), NFI (0.926) and TLI (0.902) are acceptable with the cut-off value. The value for RMSEA (0.089) is bit exceeds to threshold value suggested by Hu and Bentler (1999), however, it was in the acceptable level. RMR (0.018) indicates better values. The normalized Chi square (8.076, P value: 0.000) is bit exceeds than the threshold. Accordingly, giving attention to all fit indices, the modified model was considered for the final model design.

The CFA was performed to the model of Attitude toward to use (A_use) and modification indices were reviewed. The covariance was identified and made connection to error term e1 to e2, error term e4 to e5 and error term e5 to e6 as the initial model was not fit well. The values after the modification show the acceptable model fit. The normalized Chi Square (4.361, P value: 0.000) is less than the threshold value. Other model fit indices values also are within the acceptable range (GFI: 0.979, AGFI: 0.956, CFI: 0.981, NFI: 0.976, TLI: 0.957, RMSEA: 0.061 and RMR: 0.018). The CFA results of the use of e-government service (Useofe-Gov) show that there is no any interconnection contact and relationships. Thus, model fit indices were not generated for this model. Nevertheless, factor loadings were concerned and it was revealed that loading were statistically significance with reaching the threshold requirement. Hence, construct validity was ensured. Factor loadings are IS: 0.69, CON: 0.71 and WU: 0.74 and these values exceed the threshold value of 0.5. Accordingly, Useofe-Gov model is acceptable for the final model.

After modifications, all constructs are acceptable to final model and variable scales for all items are suitable enough to proceed with further analysis. The summary of results is shown in Table 2. Thus, the next step is to analyze the proposed model with all constructs.

**Table 2:** CFA results and AVE and CR values

Constructs	Chi square	GFI	AGFI	CFI	NFI	TLI	RMSEA	RMR	AVE	CR
Trustworthiness	2.098	0.996	0.986	0.996	0.993	0.991	0.035	0.007	0.55	0.85
Perceived usefulness	4.756	0.970	0.944	0.996	0.959	0.951	0.065	0.025	0.52	0.91
Perceived ease of use	8.076	0.952	0.910	0.935	0.926	0.902	0.089	0.018	0.50	0.89
Attitude toward to use	4.361	0.979	0.956	0.981	0.976	0.957	0.061	0.018	0.59	0.92
Use of e-government services	Values for the model fit indices were not generated due to saturation Hence factor loadings were concerned (IS-0.69, CON-0.71, WU-0.74)								0.64	0.84

## Model fit for proposed model

With above discussion, the scale items which are used to measure each construct was tested separately. In doing so, it was able to identify that initial separate constructs are not fitted well and accordingly, modifications were done as suggested by the modification indices of each CFA outcome. Afterwards, it is essential to test all constructs which are going to consider for the final model simultaneously to get a good fit for the proposed model.

Consistency between research results and the theory is shown by a good fit model. A good fit is determined by various fit indices suggested by scholars (Hair et al. 2006; Hu and Bentler 1999). Normalized Chi square, RMSEA and GFI are considered as most important fit indices which show a good fit for a model (Hair et al. 2006). The CFA was performed for the proposed model and the CFA results reveal that proposed model can be accepted. The normalized Chi square is 2.769 (P value: 0.000), which is less than the threshold value of five. The RMSEA value is 0.044, which is less than 0.08. Hu and Bentler (1999) have shown that if RMSEA value is less than 0.06, the model fit is good. Next important fit index is GFI; value for the index is 0.906, which is in the acceptable range. Furthermore, the study concerned other fit indices too for the final assessment of the proposed model. The fit indices values of NFI: 0.890 and AGFI: 0.889 show acceptable values which is very closer to threshold value of 0.9. The value for CFI: 0.926 and TLI: 0.919 exceed the cut-off value of 0.9. Apart from those indices, root mean square residual (RMR) index shows a good value of 0.32. This index value ranges from 0 to 1, with a value of 0.08 or less indicates an acceptable model.

**Table 3:** CFA results for the whole model

	Chi square	GFI	AGFI	CFI	NFI	TLI	RMSEA	RMR
Overall model	2.769	0.906	0.889	0.926	0.890	0.919	0.044	0.032

**Fig. 3:**
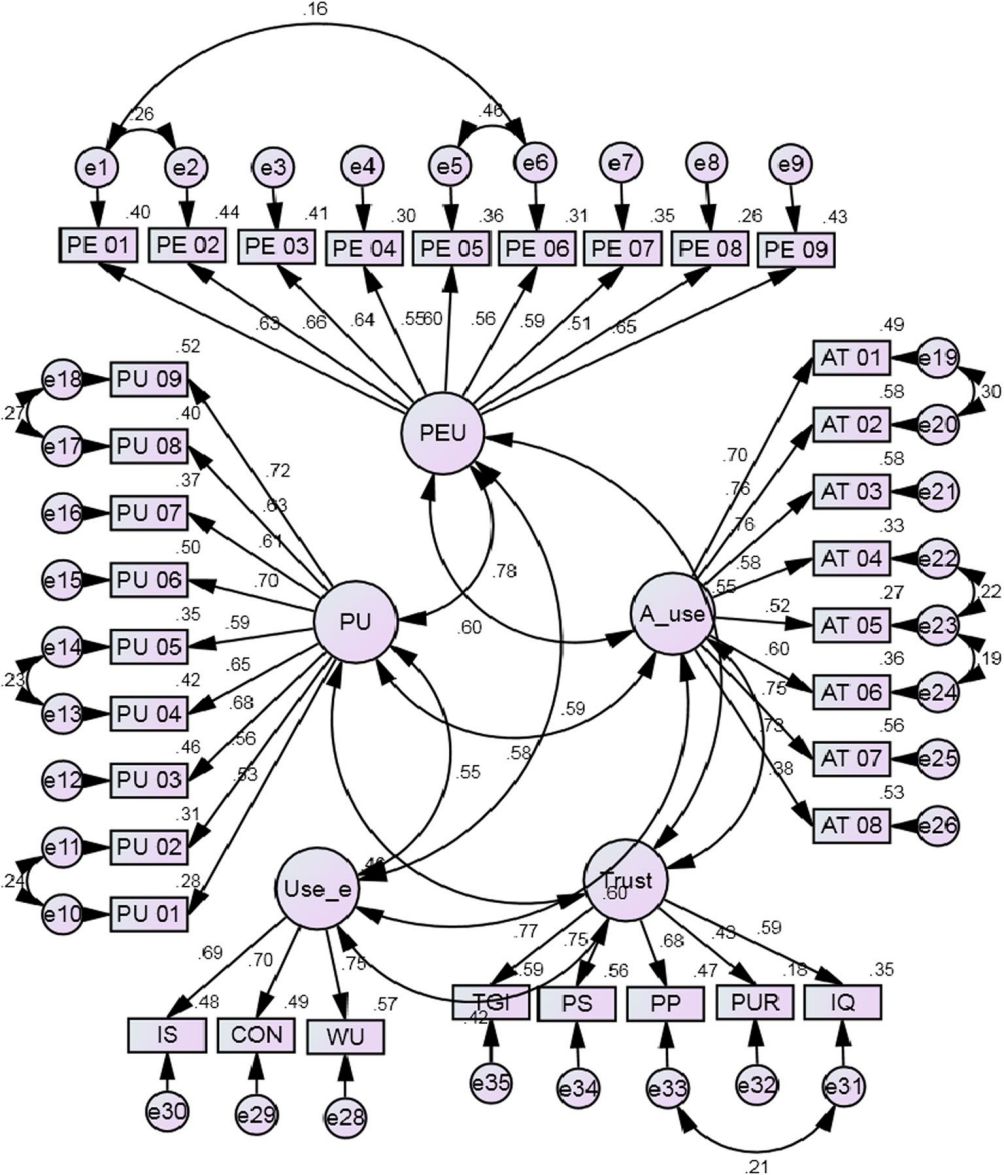
Modified proposed model

Accordingly, comparing all the relevant fit indices values shown in Table 3, the proposed model shows a good fit and proposed model is accepted. The modified proposed model is illustrated by the Fig. 3 and can be used for further structural analysis.

## Conclusion

In contrast to e-government adoption studies, research in e-government adoption in Sri Lankan context is few and it needs to be explored further. Thus more empirical studies are required to understand the e-government initiatives in Sri Lanka. E-government projects are planed and implemented expecting more benefits. But, as so far concerned, literature reveals that most of the e-government projects were not successful due to various issues. One of the issues causing the less adoption and use of e-government service is trustworthiness. Thus, the objectives of this study were to identify the antecedent conditions of trustworthiness and propose a conceptual framework that can serve as a base model which is worth to assess the influence of the trustworthiness for the use of e-government services in Sri Lanka. In the modified, tested and accepted model, five dimensions of the trustworthiness (trust in government and internet, perceived security, perceived privacy, perceived uncertainty/risk and information quality) were considered and make the model broader from the perspective of trustworthiness. The results of the CFA suggested a well fitted model and fit indices recommend the model to apply in the adoption and use of e-government services from the perspective of trustworthiness. Furthermore, the tested model can be applied in a similar context in other countries those having issues related to adoption and use of e-government services. This research was limited to propose a model and the relations between factors are yet to be analyzed using structural model. Thus, future task would be assessing the relationships among the factors. The outcome will be useful for all stakeholders and give opportunity for better understanding and take remedies to existing issues pertaining to e-government initiatives and successful implementation.
